# Stimulation of HERG Channel Activity by β-catenin

**DOI:** 10.1371/journal.pone.0043353

**Published:** 2012-08-15

**Authors:** Carlos Munoz, Ambrish Saxena, Tatsiana Pakladok, Evgenii Bogatikov, Jan Wilmes, Guiscard Seebohm, Michael Föller, Florian Lang

**Affiliations:** 1 Department of Physiology, University of Tübingen, Tübingen, Germany; 2 Biochemistry I, Ruhr University Bochum, Bochum, Germany; 3 Campbell Family Institute for Breast Cancer Research, Ontario Cancer Institute, University Health Network, Toronto, Ontario, Canada; University of South Florida College of Medicine, United States of America

## Abstract

The multifunctional protein ß-catenin governs as transcription factor the expression of a wide variety of genes relevant for cell proliferation and cell survival. In addition, ß-catenin is localized at the cell membrane and may influence the function of channels. The present study explored the possibility that ß-catenin participates in the regulation of the HERG K^+^ channel. To this end, HERG was expressed in *Xenopus* oocytes with or without ß-catenin and the voltage-gated current determined utilizing the dual electrode voltage clamp. As a result, expression of ß-catenin markedly upregulated HERG channel activity, an effect not sensitive to inhibition of transcription with actinomycin D (10 µM). According to chemiluminescence, ß-catenin may increase HERG channel abundance within the oocyte cell membrane. Following inhibition of channel insertion into the cell membrane by brefeldin A (5 µM) the decay of current was similar in oocytes expressing HERG together with ß-catenin to oocytes expressing HERG alone. The experiments uncover a novel function of APC/ß-catenin, i.e. the regulation of HERG channels.

## Introduction

The multifunctional protein ß-catenin is involved in the regulation cell proliferation and tumor growth [1], [2]. ß-catenin further participates in the physiology and pathophysiology of cardiac hypertrophy [3]–[6]. ß-catenin degradation is initiated following phosphorylation by glycogen synthase kinase 3 beta (GSK3ß) [7], [8], a kinase counteracting cardiac hypertrophy [9], inhibiting apoptosis and fibrosis and thus increasing cardiac contractility [10], [11]. Overexpression of ß-catenin is followed by dilated cardiomyopathy and premature death [12].

ß-catenin may enter the nucleus and stimulate the expression of several genes important for cell proliferation [13], [14]. ß-catenin-stimulated genes include the serum and glucocorticoid inducible kinase SGK1 [15], [16], which is required for cardiac fibrosis following mineralocorticoid excess [17]. ß-catenin may further be localized at intercalated disks [18], bind to cadherin [19] and play a role in the regulation of gap junctions [20].

ß-catenin has been shown to interact with and/or modulate the activity of large-conductance Ca^2+^-activated K^+^ channels [21], kainate receptors [22], Kv1.5 K^+^ channels [23] and KCNQ1/KCNE1 K^+^ channels [24]. Moreover, ß-catenin has been shown to colocalize with and up-regulate the Na^+^/K^+^ ATPase [25]. The interaction with ß-catenin may recruit channels to cadherin/catenin complexes leading to stabilization of the channel proteins [22].

The present study explored the possibility that ß-catenin participates in the regulation of the human ether-a-go-go (HERG, Kv11.1) channel, which is critically important for the shaping of the cardiac action potential [26], [27] and by the same token is essential for the proliferation of some tumor cells [28], [29]. HERG is downregulated in cardiac hypertrophy [30]. To this end, HERG was expressed in *Xenopus* oocytes with or without the expression of ß-catenin. The results reveal that the coexpression of ß-catenin leads to marked upregulation of HERG activity by enhancing the plasma membrane abundance of the channel protein.

**Figure 1:**
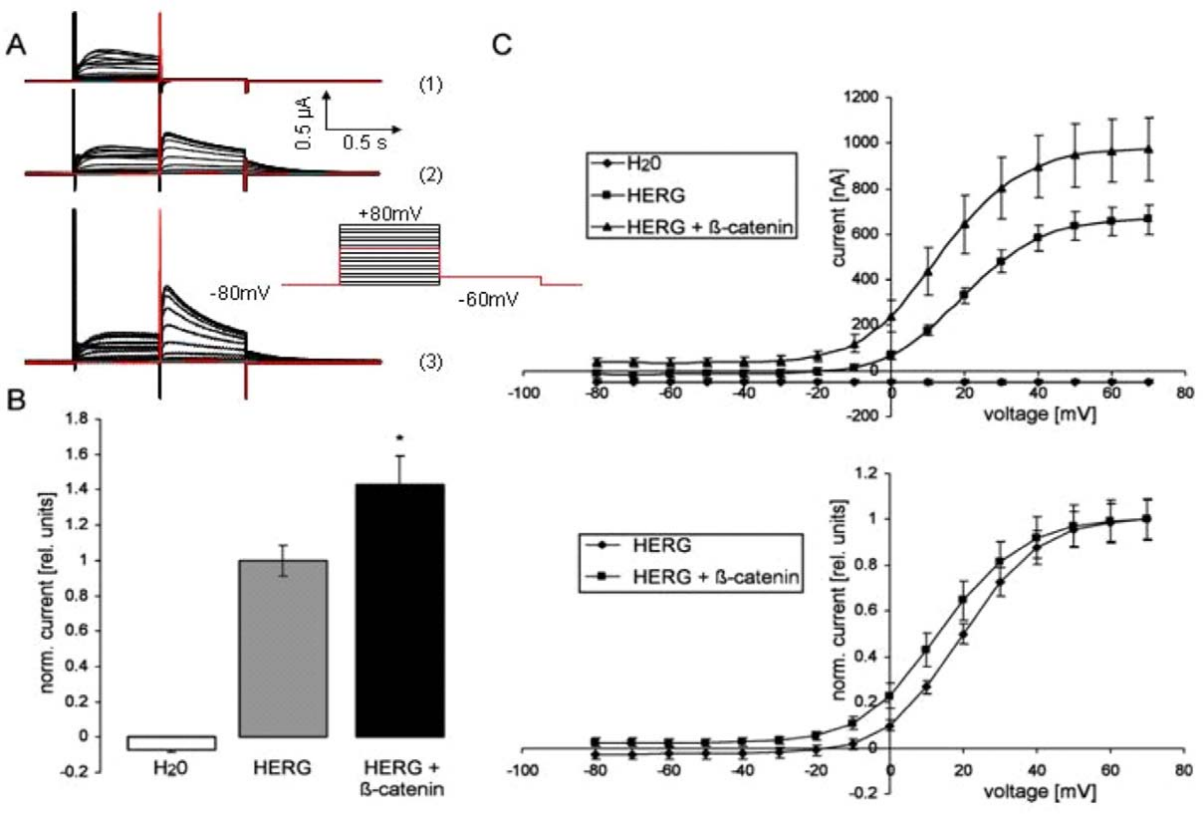
ß-catenin increases HERG current. **A.** Original tracings recorded in oocytes injected with H_2_0 (1), with cRNA encoding HERG (2) or HERG coexpressing ß-catenin (3). The oocytes were depolarized from −80 mV holding potential to different voltages followed by a 500 ms pulse to −60 mV evoking outward tail currents. The small insert displays the applied voltage protocol. **B.** Arithmetic means±SEM (n = 8–11) of the normalized outward tail current following a depolarization to +80 mV recorded in oocytes injected with H_2_0 (left bar), with cRNA encoding HERG (middle bar) or with RNA encoding HERG and ß-catenin (right bar). * indicates statistical significance (p<0.05) from the absence of ß-catenin cRNA. **C.** IV curves of outward tail currents illustrated in B (upper panel) and IV curves of outward tail currents following normalization to the maximal tail current of the respective group (lower panel).

## Materials and Methods

### Experiments in *Xenopus* Oocytes

For generation of cRNA, constructs were used encoding human β-catenin [25], human truncated mutant β-catenin^1–530^ [31], HERG channel [32] and N-cadherin [33].

For voltage clamp analysis, *Xenopus* oocytes were prepared as previously described [34]. Where indicated, oocytes were injected with water or 10 ng cRNA encoding β-catenin, truncated ß-catenin^1–530^ and/or N-cadherin and on the same day with 7.5 ng cRNA encoding HERG. Standard two electrode voltage clamp recordings were performed 3 days after HERG injection [35]. Oocytes were superfused continuously with ND-96 buffer containing (mM): NaCl 96, KCl 2, CaCl_2_ 1.8, MgCl_2_ 1 and HEPES 5 (pH 7.4 with NaOH). Pipettes were filled with 3 M KCl and had resistances of 0.5–1.0 MΩ. Experiments were performed with a Geneclamp 500B amplifier (Axon Instruments, Union City, CA, USA) and a Digidata 1322A interface (Axon Instruments, Union City, CA, USA). Data acquisition was achieved with pCLAMP 9.02 (Axon Instruments, Union City, CA, USA).

Where indicated, the experiments were performed in *Xenopus* oocytes treated with 10 µM actinomycin D one day before measurement to disrupt gene transcription. To discriminate between alterations of insertion and retrieval of HERG channel protein from the plasma membrane, the insertion was inhibited by brefeldin A [36], where indicated. In those experiments, the oocytes were preincubated in the presence of Brefeldin A (Sigma, Schnelldorf, Germany)one day before measurement at a concentration of 5 µM. Tail currents, which indicate, what fraction of the channels are open following a transient voltage step, were taken as a measure of channel activity [37].

**Figure 2:**
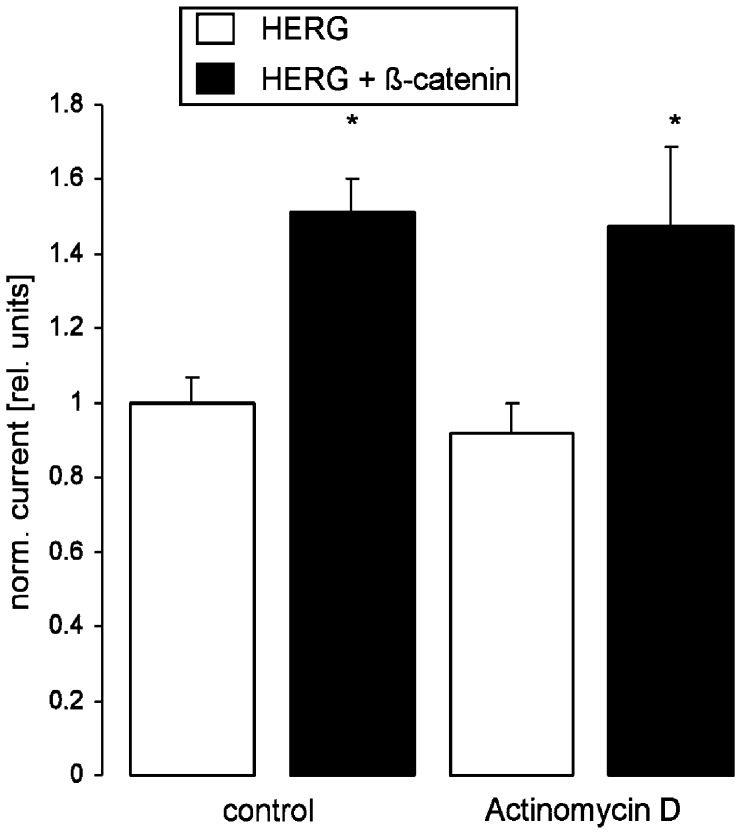
The effect of ß-catenin on HERG currents is not modified by actinomycin D. Arithmetic means±SEM (n = 9–13) of the normalized outward tail current following a depolarization to +80 mV recorded in oocytes injected with cRNA encoding HERG (white bars) or with RNA encoding HERG and ß-catenin (black bars), incubated for 24 hours without (left bars) or with (right bars) 10 µM actinomycin D prior to the measurement. *indicates statistical significance (p<0.05) from the absence of ß-catenin cRNA.

**Figure 3:**
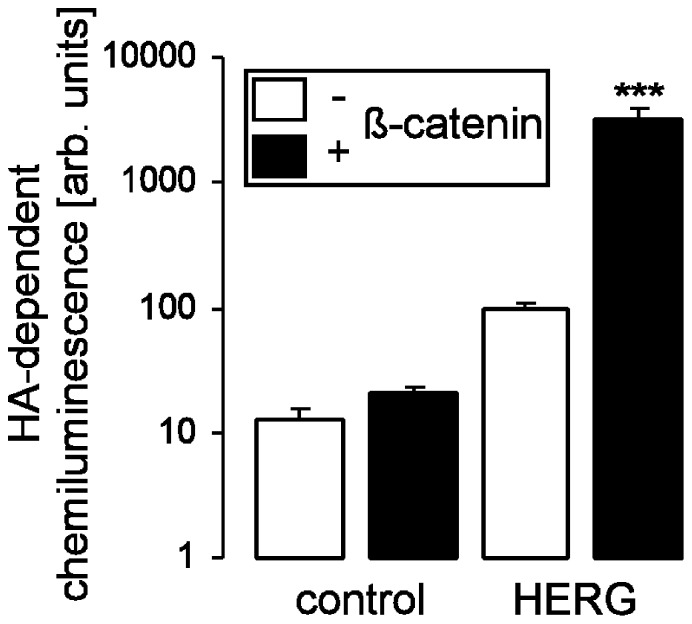
ß-catenin increases the surface abundance of HERG. Arithmetic means±SEM (n = 19–56) of the normalized HA-dependent surface chemiluminescence of oocytes injected with H_2_0 (1^st^ bar), with cRNA encoding ß-catenin (2^nd^ bar), encoding HERG (3^rd^ bar) or encoding both, HERG and ß-catenin (4^th^ bar). ***indicates statistical significance (p<0.001) from the absence of ß-catenin cRNA.

To determine HERG cell surface expression by chemiluminescence [38], defolliculated oocytes were first injected with 7.5 ng cRNA encoding either HERG-HA or 10 ng cRNA encoding β-catenin. After 3 days of incubation oocytes were incubated with 1 µg/mL primary rat monoclonal anti-HA antibody (clone 3 F10, Roche, Mannheim, Germany) and subsequently with secondary, HRP-conjugated goat anti-rat IgG (H&L) antibody (1∶1000, Cell Signaling Technology, MA, USA). Individual oocytes were placed in 96 well plates with 20 µl of SuperSignal ELISA Femto Maximum Sensitivity Substrate (Pierce, Rockford, IL, USA), and chemiluminescence of single oocytes was quantified in a luminometer (Walter Wallac 2 plate reader, Perkin Elmer, Juegesheim, Germany) by integrating the signal over a period of 1 s [39]. Results display normalized relative light units.

### Statistical Analysis

Data are provided as arithmetic means ± SEM; n represents the number of oocytes or cells investigated. All oocyte experiments were repeated with at least three batches of oocytes; in all repetitions, qualitatively similar data were obtained. As different batches may yield different expression levels and currents, comparisons have always been made within the same batches of oocytes. All data were tested for significance by using ANOVA. Results with P<0.05 were considered statistically significant.

**Figure 4:**
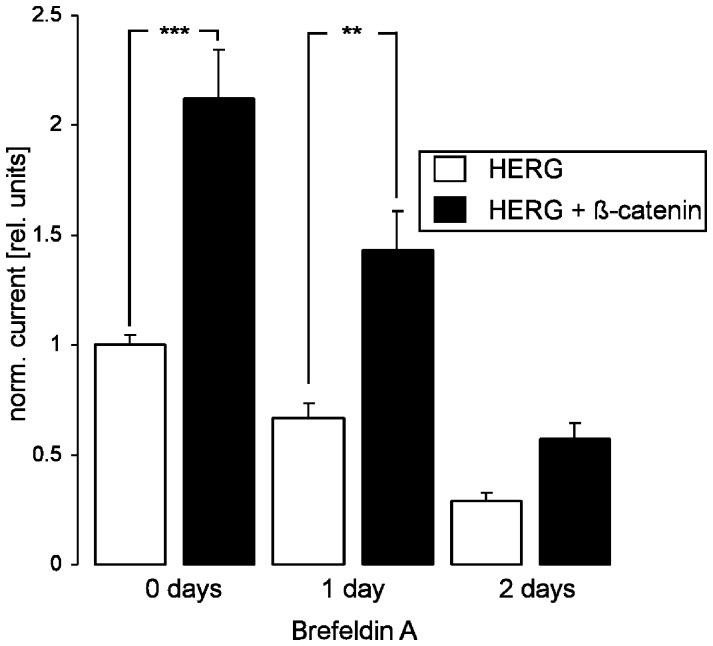
The effect of Brefeldin A on ß-catenin-stimulated HERG currents. Arithmetic means±SEM (n = 15–19) of the normalized outward tail current following depolarization to +80 mV recorded in oocytes injected with cRNA encoding HERG (white bars) or with cRNA encoding HERG and ß-catenin (black bars), prior to (0 days) and following incubation for 24 hours (1 day) or 48 hours (2 days) with 5 µM Brefeldin A prior to the measurement. **, ***indicate statistical significance (p<0.01, p<0.001) from the absence of ß-catenin cRNA.

## Results

### Coexpression of ß-catenin Increased HERG Current

In oocytes injected with cRNA encoding HERG but not in water-injected oocytes depolarization from −80 mV holding potential to different voltages followed by a 500 ms pulse to −60 mV evoked outward tail currents (Fig. 1AB). The additional expression of ß-catenin was followed by a marked increase in the tail current (Fig. 1AB). Fig. 1C summarizes the current voltage relationship of HERG currents with or without coexpression of ß-catenin. The amplitude of the peak tail current was plotted as a function of the preceding test potential. The absolute current values were markedly upregulated by coexpression with ß-catenin. The tail currents that were normalized to the maximum peak tail current of the respective group to investigate kinetics were not significantly modified by the coexpression of ß-catenin, i.e. the voltage evoking half maximal peak tail currents was similar in HERG expressing oocytes with or without additional expression of ß-catenin. The effect of ß-catenin was not blunted by treatment of HERG-expressing *Xenopus* oocytes with 10 µM actinomycin one day before measurement to prevent ß-catenin-dependent gene expression (Fig. 2).

**Figure 5:**
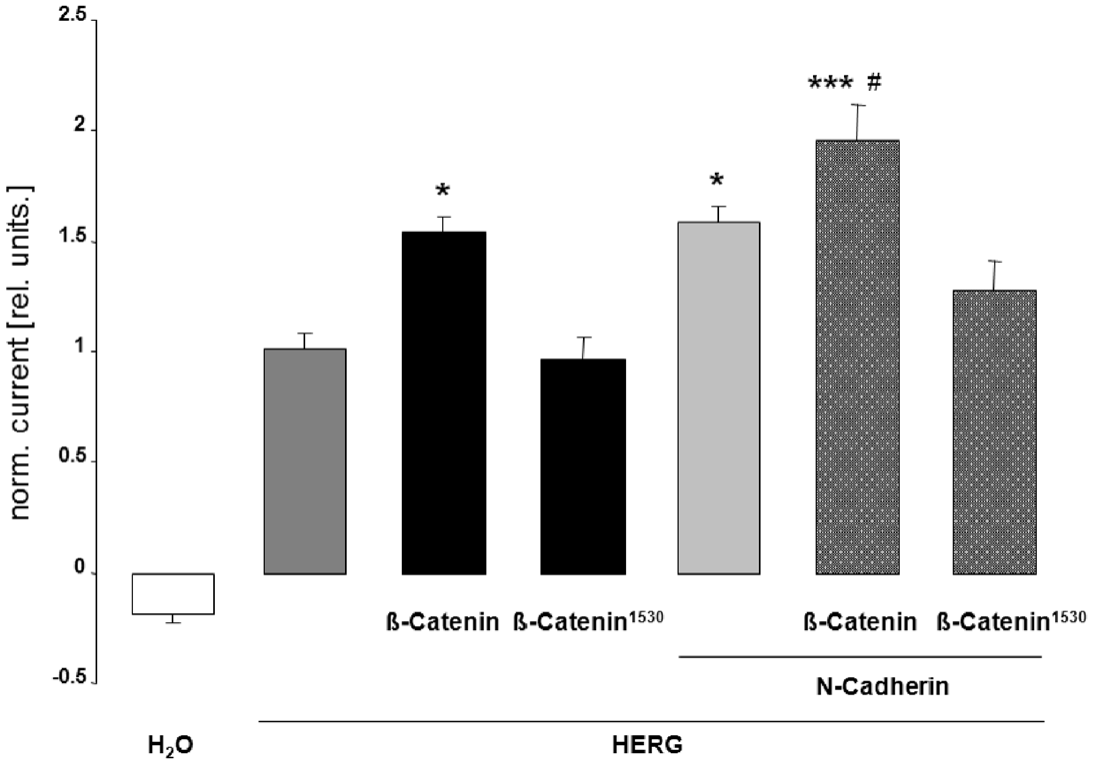
HERG currents are insensitive to truncated ß-catenin^1–530^ and stimulated by N-cadherin. Arithmetic means ± SEM (n = 10–20) of the normalized outward tail current following depolarization to +80 mV and recorded in oocytes injected with water (white bar), or with cRNA encoding HERG (dark grey bar) with ß-catenin (first black bar) or with the truncated mutant ß-catenin^1–530^ (second black bar), without and with co-expression of N-cadherin (dotted dark grey bars). *, ***indicate statistical significance (p<0.05, p<0.001) from the absence of HERG cRNA, # (p<0.05) indicates statistical significance from the absence of N-cadherin.

### ß-catenin Increased the Surface Expression of HERG

Binding of a specific antibody and subsequent determination of HA-dependent surface chemiluminescence was employed to determine the effect of ß-catenin on oocytes expressing HA-tagged HERG. As shown in Fig. 3, expression of HERG led to a profound increase in HA-dependent chemiluminescence. More importantly, coexpression with ß-catenin significantly increased the abundance of HERG channels in the plasma membrane as revealed by an elevated HA-dependent surface chemiluminescence signal (Fig. 3).

To discriminate between increased HERG protein insertion into and delayed retrieval of HERG protein from the plasma membrane, additional experiments were performed in the presence of Brefeldin A (5 µM) which prevents the insertion of novel proteins advancing from the Golgi apparatus into the cell membrane [36]. As illustrated in Fig. 4, in the presence of brefeldin A the current decreased in *Xenopus* oocytes expressing HERG together with ß-catenin as fast as in *Xenopus* oocytes expressing HERG alone. This observation discloses that ß-catenin does not delay the retrieval of HERG protein from the membrane.

### The Effect of ß-catenin on HERG Current was Abrogated by ß-catenin Truncation and Mimicked by Coexpression of N-cadherin

To determine, whether the effect on HERG channels requires full-length ß-catenin, HERG channels were expressed in *Xenopus* oocytes with or without additional expression of the truncated mutant ß-catenin^1–530^. As shown in Fig. 5, truncation abrogated the effect of ß-catenin on HERG channel activity. Since truncation of ß-catenin disrupts the binding of ß-catenin to N-cadherin [40], additional experiments were performed to elucidate the effect of N-cadherin. As illustrated in Fig. 5, the additional coexpression of N-cadherin further upregulated channel activity in HERG-expressing *Xenopus* oocytes.

## Discussion

The present observations uncover a completely novel function of ß-catenin, i.e. the regulation of HERG channel activity. ß-catenin did not alter HERG channel kinetics but apparently increased the HERG protein abundance within the cell membrane. The effect is apparently not due to delayed retrieval of channel protein from the cell membrane but may result from enhanced insertion of HERG channel protein into the cell membrane.

The effect of ß-catenin did not result from its function as a transcription factor, as it was not significantly modified by suppression of transcription with actinomycin. Moreover, the experiments were performed following heterologous HERG expression. Thus, the functional expression of HERG did not depend on genomic regulation of the channels. The present observations did not define the mechanisms underlying ß-catenin sensitivity of HERG channel activity. ß-catenin has previously been shown to interact with channel proteins [21], [22] leading to recruitment of channels to cadherin/catenin complexes with eventual stabilization of the channel proteins [22]. According to the present study, the effect of ß-catenin was mimicked by N-cadherin and disrupted by truncation of ß-catenin.

The ß-catenin-sensitive HERG channel activity may, at least in theory, impact on the cardiac action potential during cardiac hypertrophy. Enhanced HERG activity was expected to accelerate the repolarization of ventricular muscle cells, shorten the action potential thus favouring reentry. Cardiac hypertrophy is facilitated by decreased activity of the glycogen synthase kinase-3 beta (GSK3ß) [9], which in turn leads to enhanced ß-catenin abundance [7], [8]. As a matter of fact, cardiac hypertrophy is paralleled by enhanced ß-catenin abundance and activity. However, HERG channels have been described to be downregulated in cardiac hypertrophy [30], [41], an effect mediated by mechanisms other than ß-catenin, such as activation of AT_1_ receptors with subsequent activation of protein kinase C linked to the PKC pathway in ventricular myocytes [42].

ß-catenin is regulated by the Wnt pathway [43], which is known to regulate cardiac development and function [20], [44]–[48]. To the extent that HERG channel activity is dependent on ß-catenin, it is regulated by the Wnt pathway. As stimulation of the Wnt pathway downregulates GSK3 and thus leads to upregulation of ß-catenin, it would be expected to upregulate HERG channel activity.

HERG channels are further implicated in the regulation of tumor growth [28], [29]. Dysregulation of the oncogene ß-catenin is in turn considered a major cause of tumor development. It is tempting to speculate that ß-catenin-sensitive regulation of HERG protein abundance in the cell membrane contributes to the dysregulation of cell proliferation in some tumor cells.

In conclusion, the present observations provide compelling evidence that ß-catenin upregulates the voltage-gated K^+^ channel HERG.
